# Supplementary material for: Complexome profiling of the Chlamydomonas psb28 mutant reveals TEF5 as an early PSII assembly factor
Source: Plant Cell. 2025 Mar 17;37(6):koaf055. doi: 10.1093/plcell/koaf055 (PMC12164590; doi:10.1093/plcell/koaf055)
Supplement: koaf055_Supplementary_Data [file koaf055_supplementary_data.zip › psb28_interactive.zip!/plots/intensity/qualityPlots/peptides/CYC2_Cre12.g522600.t1.2.html]

### Cre12.g522600.t1.2

Trivial name: CYC2  
SeqCoverage [median]: 54.50  
Mass: 11.96 kDa  
  
 

| Sequence | MissedCleavage | Mass [Da] | wtIntensity | muIntensity | Score |
| --- | --- | --- | --- | --- | --- |
| ADLIAYLK | 0 | 905.52 | 46840.44 | 26890.36 | 151.29 |
| GGGHKQGPNLGGLFGR | 1 | 1550.81 | 471.99 | 0.00 | 45.86 |
| MVFAGLK | 0 | 764.43 | 885.73 | 0.00 | 94.67 |
| MVFAGLKKPEER | 2 | 1403.76 | 1299.21 | 0.00 | 78.69 |
| QGPNLGGLFGR | 0 | 1114.59 | 4352.68 | 10144.79 | 171.75 |
| STFAEAPAGDLAR | 0 | 1304.64 | 37011.01 | 35743.81 | 198.62 |
| VSGTAAGFAYSK | 0 | 1157.57 | 43862.85 | 24064.65 | 196.88 |
|  |  |  | 134723.9 | 96843.6 |  |
